# Erosion

**Published:** 1891-02

**Authors:** William H. Trueman

**Affiliations:** Philadelphia


### EROSION.[1]

BY WILLIAM H. TRUEMAN, D.D.S., PHILADELPHIA.

Abrasion, erosion, and caries are three peculiar and distinctive processes by which is wrought, through a progressive and gradual loss of tissue, the destruction of the human teeth. They differ in their etiology, probably, as widely as they do in appearance and general characteristics, and yet they seem to be in many cases so linked together, and to follow each other so closely, that it is difficult to assign the injury done to either factor alone. This is not, however, always the case. The injury may be purely a mechanical abrasion, or it may be that erosion or caries exists alone, and the appearances presented are characteristic and unmistakably those of the destructive process concerned.

In looking over the literature of this subject, I find several papers upon erosion devoted almost exclusively to the various forms of receding gums and sensitive dentine associated therewith. I cannot conceive that they have anything in common. Erosion may exist at the necks of teeth, and frequently does, where the gums are quite normal. I have at times found it entirely below the gum-line, with the gum-tissue normally adherent at the lower margin of and completely covering the eroded cavity. While there is usually associated with it, owing to the loss of tissue, an abnormal sensitiveness of the teeth, this is not constant; and, on the other hand, there may be extensive recession of the gum and excessive sensitiveness of the exposed necks of the teeth without the slightest erosion. While these several conditions may jointly exist they are not inseparably associated, but are in origin and history distinctive and independent abnormal conditions.

Until within a few years the etiology of erosion was a closed book. The only theories advanced were either so greatly at variance with frequently-observed facts, or so indefinite, that they received, and were entitled to, but little attention.

Until very recently equally unsatisfactory were the various theories to explain the cause of dental caries. Some were absurdly preposterous; others were reached by close observation and well-considered reasoning, and contained much that we now recognize as true. Not, however, until Dr. W. D. Miller, of Berlin, Germany,

---

[1] Read at the annual meeting of the New Jersey State Dental Society, July, 1890.

published the results of a long series of carefully-conducted experiments was the real cause of dental caries known. Not until he accomplished it had any investigator, by the application of his own theory, produced dental caries out of the mouth precisely the same as that we so constantly see in the mouth. This was the crowning act of Dr. Miller's patient and persistent investigations. Others before him had demonstrated the presence of fermentable matter in the mouth, the presence of various microscopic fungi there, and in carious cavities in the teeth; but it was reserved for him to demonstrate how these two long-observed factors worked together to produce the effect so frequently noted, and to conclusively solve the perplexing problem. We may not as yet have reaped much practical benefit from the exact knowledge thus acquired, but, knowing the enemy with which we have to contend, it must in the near future make our labors for the arrest and suppression of dental caries more successful and satisfactory. The solving of this problem has encouraged other investigators to step from the beaten track, and we may now hope that persistent and well-directed efforts will, step by step, wrest from erosion the secret of its birth.

Abrasion, which in appearance so nearly simulates and which is so often confounded with erosion, is a purely mechanical removal of tooth-tissue. Its effects are seen more frequently upon the cutting and masticating surfaces; it is, indeed, the natural process by which the cutting edges of the incisors are, after their eruption, wrought to their normal shape, and by which the masticating surfaces of the bicuspids and molars are made to closely articulate. The amount of tissue thus removed, when the dental development is quite normal, is exceedingly small; and although this abrasion ordinarily progresses slowly yet steadily as the years roll on, it does not usually, when confined to these surfaces, entail any serious complications. While its effects are most marked upon the masticating, they are also seen in a less degree upon the approximal surfaces of all the teeth that are in close contact, especially in the middle and later periods of life, when the teeth are apt to become less rigid in their positions. It is here, too, that it is apt to be complicated with erosion and caries, and is largely responsible for approximal cavities developed at this time in dentures that heretofore have been perfectly sound. At this period of life there is usually a natural and normal consolidation or shrinkage; in many cases, indeed, an abnormal recession of gum-tissue, that leaves between the teeth spaces favoring the lodgement of effete matter, or in which the oral secretions are in various ways retained and there undergo a change that renders them destructive of tooth-tissue. This leads in some cases to the formation of carious cavities; in others it gives place to the more rapid process of tooth-destruction,—caries. This is most frequently observed in, but is not confined to, the posterior teeth. As this observed fact has an important bearing upon our subject, let us examine it a little more closely, confining our attention to the molar teeth.

The approximal surfaces of well-formed molar teeth are so rounded that they present but little surface in contact, this and the gum-tissue, closely filling the space below the line of contact, prevents the undisturbed accumulation of effete matter. By the process of abrasion these rounded surfaces are flattened, and those in contact are thereby enlarged; this and the recession of gum-tissue favors the retention of oral secretions, etc., and the process of erosion begins. This continues slowly until, by loss of tissue, a comparatively large space is formed. This space usually assumes upon each tooth a concave form, and is thus better fitted to collect and retain fermentable matter, and favors the location of tooth-destroying germs which augment and continue the destructive process. This loss of tissue by erosion may quickly open on either tooth a structural defect that furnishes these germs a place for lodgement, and we have quickly formed a large and penetrating cavity. I am well satisfied that this is substantially the history of many approximal cavities that develop in the later periods of life, and I am equally well satisfied that in their formation each of these three destructive processes—abrasion, erosion, and caries—plays a part.

The distinctive characteristics of erosion and the very marked dissimilarity between it and caries, both in appearance and origin, were early noted and its varied modifications accurately described, but beyond suggesting that it was a real and actual solution of tooth-tissue, few writers ventured to account for its presence. But a little observation demonstrated positively that it had a widely-differing origin from abrasion, which in physical characteristics it so closely resembled. While generally admitting that it was a solution of tooth-tissue, that the destructive agent was an acid, the question naturally arose, why should its destructive action be so sharply localized, and why should it exhibit throughout its course rigid and sharply-defined lines? These questions have always been the stumbling-blocks in the way of a tenable theory.

The peculiar and characteristic appearances of abrasion and erosion are so closely similar that in many cases it is, without a history of the case, impossible to distinguish between them. The lower bicuspid tooth here shown, from the mouth of a patient aged about sixty years, was, except a slight recession of the gum, quite normal. Several of the anterior teeth then had eroded cavities at the gum-margin which have since enlarged, and those teeth, while still retained, have suffered from erosion to a greater extent than this has. I first noticed the erosion upon this tooth shortly after the extraction (made necessary by the recession of the gums and consequent loosening) of several teeth posterior to it, and in six or eight years it progressed to the condition now seen. While in this case the erosion is more marked upon the labial surface, extending two-thirds through the tooth, it is seen also upon the lingual surface, and in a less degree upon both approximal surfaces at the neck of the tooth. I have seen cases closely resembling this where the tooth has been encircled by a narrow clasp supporting a denture, the effect being due to abrasion, or, more probably, abrasion and erosion combined. This tooth, however, has never been clasped, and the injury done is entirely due to erosion.

In illustration of the close resemblance of erosion and abrasion I present this cast of a denture, the first lower molars of which on either side have lost at least two-thirds of their length; the teeth have been gradually reduced from the masticating surface and have as gradually elongated to meet the opposing teeth. This has all taken place, the patient informs me, and my own recollections confirm it, within the last eight years. These two molars are the only teeth so affected in that mouth. It is probable that there was upon their masticating surfaces, when erupted, a malformation of the enamel, there has been no loss of tissue by caries. On the left side, except that the loss is almost entirely upon the lower tooth, the occlusion is such that we might attribute it to abrasion. On the right side, however, it is impossible for the opposing tooth to reach the worn surface of the lower tooth in any position that the jaw may assume. The patient states that she first noticed the wearing of this tooth shortly after the loss of a molar posterior to it. She was then about thirty years of age. The inner integument of the cheeks is quite voluminous, and rests closely against the teeth. On the right side, owing to the position of the upper posterior teeth and the loss of the two lower molars, the integument of the cheek completely covers the masticating surface of the first molar when the mouth is at rest. Have we here an example of erosion upon one side of the mouth and of abrasion upon the other, or is the loss of substance in both teeth due to erosion? I have no doubt that the enamel upon the masticating surfaces of both teeth was pitted and defective; it is to be stated, however, that no unusual wear was noticed until after the teeth had been in position many years, not, indeed, until the loss of adjacent teeth permitted the soft tissues to encroach upon and overlay the masticating surfaces. Within a year or two there has developed a slight erosion near the necks of several of the anterior teeth that is slowly progressing.

In another case, the upper molar has lost considerable of its masticating surface, and the posterior portion of the same surface of the lower molar immediately under it has also been lost; the loss from the lower tooth extends only so far as it is covered by the upper molar, so that the masticating surfaces of the two teeth form with each other a wide angle, the apex at the line of the anterior approximal surface of the upper molar, with a space between them of some three-eighths of an inch at the posterior approximal line. In this mouth several of the teeth are of defective structure, as shown by a brown discoloration. Both of the teeth we are considering were so marked. There has been upon them no caries, they are not now, nor have they ever been, filled. There is at the junction of the enamel and dentine a sharply-marked depression, otherwise the surfaces are, although somewhat discolored, smooth and perfectly polished. In this case loss of the second lower molar has allowed the integument of the cheek to encroach upon the masticating surfaces of both teeth; this and the defective tissue seem to have been the localizing cause of the erosion, for we may in this case unquestionably attribute the loss of tooth-tissue to that alone.

In the next case we have upon the labial surface of a lower molar an eroded cavity closely resembling those found so frequently upon the labial surfaces of the anterior teeth. The enamel at the lower margin of this cavity formed an extremely sharp, knife-like edge that irritated the patient's cheek. It has been suggested that the initial injury in this case may have been a fracture or scaling off of a portion of the enamel. I am disposed, however, to consider it a case of erosion.

The next case is much more complicated. The patient, aged about fifty-three, has been a tobacco-chewer. As shown by the casts, the line of occlusion is very irregular, and yet by some means the upper and lower teeth have been mutually acted upon, so that the masticating surfaces are in almost perfect contact; indeed, so perfectly do they fit and interlock the one into the other that when the mouth is closed there is not and cannot be between them the slightest rubbing motion. This is well shown by a narrow ridge upon the cutting edges of both upper centrals immediately over the interspace between the closely-crowded lower incisors opposed to them. The uneven line of occlusion in this case is in striking contrast to the perfectly level surface so frequently seen in dentures worn down by mastication. In this instance there is possibly a sliding motion upon either side as the teeth approach and before they are completely closed; this may have contributed to the very marked loss of tissue. It has been also suggested that this loss of tissue may have been due to abrasion by impact, a wearing away of tooth-tissue by much the same process as that by which granite is wrought into shape, the surface being crushed or abraded by repeated blows of a hammer or of a blunt chisel. Originally, it has been suggested, the loss of tissue began by simple abrasion due to mastication until from some teeth the enamel was entirely worn from the surfaces in contact. The dentine being less resistant and wearing away more rapidly, tended to eventually bring about the uneven occlusion. This restricted the sliding motions of the lower jaw, and the wearing being continued by impact has produced the condition now seen. While giving this plausible theory due weight, I am not disposed to attribute the effect entirely to so slow a process. Undoubtedly it has been going on for years; eight years ago, when I first noticed it, the loss of tissue and the tendency to uneven occlusion was quite marked. I have been impressed, however, by the rapid progress made the last two years; that we have now another factor to consider, and that the destruction commenced by abrasion is now being much more rapidly continued by erosion. Were it abrasion only, I see no reason why it should progress so much more rapidly now than it did a few years ago, the difference in density between the enamel that has been lost and the dentine that is now being worn away not being sufficient to account for it.

These cases are not presented as typical cases of either abrasion or erosion; they all come near the border line; and I question very much that, if shown the teeth extracted from the mouth, the most experienced among us would venture to say whether the loss of tissue was due to abrasion or erosion. In each case I have little question but that the position and appearance of that portion of the teeth which has suffered would be considered sufficient evidence of abrasion. I think you will agree with me, however, that an examination of the casts would lead to a different conclusion. As part of the history of these cases we have the observed fact that the integument of the cheek when the mouth was at rest lay in close contact with that portion of the tooth which has suffered. It is very possible that there may have been a predisposing cause that has assisted to more sharply localize the injury, a cause that may have been, indeed, the initial injury without which the loss of tissue would not have occurred. Be that as it may, undoubtedly this close contact of the integument of the cheek and the tooth has been the passive agent which has held in contact with the tooth the real and active cause of the erosion. I have no question but that in many cases the same destructive agent is in like manner assisted by the contact of occluding surfaces.

I have chosen, in treating this subject, to first trace the close resemblance in the effects produced by abrasion and by erosion, not so much on account of their, at times, close association, but rather from a strong impression that this resemblance may be, in the study of the etiology of erosion, of more importance than is at first seen. To my mind it conclusively proves that, whatever may be the destructive agent concerned in erosion, it is a solvent of tooth-tissue locally applied to, and generated at, the point effected, and excludes the idea that it is due to any general condition of the mouth, or any special or peculiar secretion. The more commonly noted cases of erosion seen at the necks, labial and buccal surfaces of the teeth are so peculiar in their form, position, and relation to each other that no other explanation seems plausible. Indeed, some observers of excellent reputation have been so impressed with the idea that the cause was strictly local, that they have insisted that it was the result of friction and purely mechanical. Others have modified this, and have suggested that it was due to a tooth-solvent assisted by friction, the latter giving to it its peculiar characteristics.

In a paper entitled "A Contribution to the Etiology of Erosion," read before the First District Dental Society, State of New York, October 5, 1886, Dr. Edward C. Kirk, of Philadelphia, makes a real advance in the study of erosion, and cleverly demonstrates its strictly local origin, crediting the suggestion to Dr. James Truman.[1] He says, "Some two years ago, during a discussion, Professor James Truman advanced the idea that erosion is probably due to the solvent action of acid mucus, secreted by the follicles situated in the labial mucous membrane, and that the disease is more active at night, from the fact that during sleep the flow of alkaline secretions from the salivary glands is arrested; hence the acid secretion of the

---

[1] *Dental Cosmos*, vol. xxix. pp. 55, 56, January, 1887.

mucous follicles is not neutralized to the same extent, if at all, as during the daytime, when the salivary secretion is active. In support of this idea Professor Truman stated that he had caused tests to be made with litmus paper of the labial mucus in the mouths of patients suffering from erosion, by the patients themselves, immediately upon wakening, and each time had found a distinctly acid reaction, and he was led therefore to believe that the disease is to be accounted for upon that ground." Dr. Kirk further says, "As tests which I had formerly made during the daytime had generally failed to show any evidence of acidity of the oral secretions, and impressed by the importance of the suggestion of Dr. Truman, I repeated the litmus test and had patients affected with erosion do the same, under the conditions proposed by him,—viz., blue litmus paper was to be placed between the incisor teeth and the lips, immediately upon wakening and before the salivary secretion has commenced to flow. The papers returned to me for examination were without an exception reddened, thus giving unmistakable evidence of having been in contact with an acid. Repetition of the tests by simply moistening the paper with the labial mucus, while the patient was in the office, generally failed to show any variation from neutrality in reaction. By making the tests with proper precautions, however, results are obtained which are decidedly different from those just mentioned. The method pursued is as follows: After the mouth has been rinsed with water, all adherent mucus and saliva is carefully wiped from the labial mucous membrane with a soft napkin. A double fold of dry napkin is then placed over the eroded teeth, and a piece of moistened blue litmus paper sufficiently large to embrace the whole area of erosion is placed upon the napkin, after which the lip is to be firmly held down upon and in contact with the litmus paper for a full minute.

"Upon removing the paper it will be found distinctly reddened, but the change of color is not uniform over the entire surface, and I desire to lay particular stress upon the appearance presented by the paper after the test is made in the manner just described. It will be found that a series of red spots, corresponding in number and position to the orifices of the labial mucous follicles, are dotted over the paper, which after a short time spread through absorption of the acid, and coalesce to form reddened areas which bear considerable resemblance in configuration to the eroded areas upon the teeth."

Dr. Kirk suggests, "That the litmus paper of commerce is not sufficiently delicate for satisfactory use in these experiments; it usually contains sufficient alkali in the coloring matter to impair its sensitiveness to very weak or dilute acids. He directs that the litmus solution prepared by the method of the United States Pharmacopœia be divided into equal portions, to one of which is added just enough dilute sulphuric acid to barely make it red; it is then mixed with the other portion. Swedish filter-paper or any pure unsized paper free from alkali, soaked in such solution and dried, will furnish litmus paper of extreme delicacy."

Dr. Kirk states, "That he invariably found that while the mucous membrane immediately overlaying the eroded area is decidedly acid, the secretion from the palatine mucous membrane will frequently be alkaline; the reaction of the mucous membrane generally being neutral or alkaline in all portions with the exception of that contiguous to the eroded teeth."

Naturally the question will arise, Is this acid condition of the mucous membrane immediately overlaying the eroded areas the cause of the erosion? May it not be that the same agent which has caused the erosion has also, by the irritation it has excited in the soft tissues, changed the character of the secretion from that portion of the mucous membrane with which it has been in contact at the same time it was in contact with the tooth. If it is the cause, and erosion is really due to the acidity of these isolated areas of acid-secreting mucous membrane, what has produced the abnormal condition at these particular spots? Is it not singular that these patches of acid-secreting mucous membrane should be situated immediately over the teeth, and at no other portion of the mucous lining of the oral cavity?

In the course of a correspondence upon this subject a few months ago, Professor James Truman suggested the probability that erosion might be due to the oral secretions while the mouth was at rest during sleep, being held in contact with the teeth by the mucus surface of the lips, cheeks, etc.; those portions of the secretions thus isolated, under certain conditions, being prone to undergo a change which rendered them a solvent of tooth-tissue. The more closely that I study the subject the more strongly am I impressed with the reasonableness of this suggested theory. During sleep, when the mouth is at rest, and the muscles are relaxed, naturally the soft tissues of the mouth at some points rest against the teeth. The peculiar confirmation of the teeth and gums, the ridges, rugæ, depressions, etc., of the mucous surfaces form pockets, enclosing, isolating from the mass of the oral secretion, and at points hold in close contact with the teeth for long periods of time portions of the oral secretions. From and in these portions of isolated oral secretions I have no question but that the tooth-solvent concerned in erosion is evolved. This, however, is but one step in solving the mystery. When we consider the peculiarities of erosion as we meet with it in daily practice, there is still much, even after the destructive agent has been identified and its origin traced, that is difficult to explain. When we fill a cavity formed by rapidly-progressing erosion only a minute fraction of an inch in width, and find that the filling completely arrests the destructive process that may have been going on with increasing rapidity for months, it is hard to realize either that the filling has materially changed the previously existing conditions and thus prevented the formation of the tooth-solvent, or that so small an area should comprise the full extent of surface with which it came in contact. In a majority of cases, in all where erosion is arrested by filling, we must have accomplished the one or the other. In some cases we fail to do either, the filling simply protecting that portion of tooth-surface and that portion only covered by it; the erosion extending outward and beyond the filling-protected area. In exceptional cases, the first manifestation of erosion covers an extended surface upon each affected tooth, and many teeth are affected at about the same period of time, but as a rule, in its first noticeable beginning the area is small, a narrow line, or a depression scarcely exceeding a small pin-head in size. At first its progress is slow, but it is exceedingly progressive. This is undoubtedly due to frequent and fresh applications of the solvent with marvellous exactness to precisely the same point. Whether the erosion is small or extended, the sharply-defined outlines characteristic of erosion is evidence of the exactness with which the solvent has been applied to and confined within a limited space. It is hardly possible that so slowly-acting a solvent would remain undisturbed long enough to produce an appreciable effect except when the mouth is at rest during sleep. We find that in many cases the formation of an eroded cavity may occupy several years, whether we consider it a slowly-progressive, continuous action or a more energetic intermittent one; in either case there has been under constantly changing conditions and at varying intervals, frequent application of the eroding agent at precisely the same spot. That this should take place seems marvellous, and yet, is it any more so than that the thousand and one muscles that our will-power controls so constantly under similar conditions act together so precisely the same that the individual is unerringly recognized by the tone of the voice or the peculiarities of expression or gait. The probability that complicated muscular movements will, under similar conditions, be accurately reproduced is well recognized in the importance given to the peculiarities of hand-writing and signatures by courts of justice. The settlement of vast estates, even the issues of life or death, have frequently been decided by the similarity in the microscopic wavering of a hair line in a signature. If in the almost automatic yet complicated act of writing, or signing a document, individual peculiarities of muscular movement are so constantly and precisely repeated, I see no reason to doubt that the integument of the mouth should in slumber assume precisely the same positions.

In conclusion: Investigation is often hampered by preconceived notions. Solution is not necessarily a chemical process. The idea that this destructive agent must be an acid having an affinity with the lime-salts of the tooth has little but tradition to support it. The little cap that we frequently see, mainly of enamel, all that remains of a baby molar, is sufficient evidence that there may be, and is, formed in the oral cavity a true solvent of tooth-tissue. I know that this effect has been produced by a normal physiological process; it is, however, none the less a true solution. The study of caries was greatly hindered by the general acceptance of the idea that it must be a chemical change, and much energy and time was wasted in the effort to isolate the acid capable of so peculiarily acting upon tooth-tissue. The process of digestion may in this connection be suggestive. The changes the food undergoes in a well-ordered stomach are to a greater extent physical than chemical, and are due far more to the active agent there secreted than to the acid associated with it. If we can settle the point that the cause of erosion is a strictly local one, that it is not due to a peculiar secretion, but to a change in a small and isolated portion of the normal oral secretion, its mechanism and chemistry will be very much simplified, and we can utilize in its study the knowledge acquired by recent investigations of the changes constantly going on within the oral cavity, largely the result of microbic energy.

